# Thoracolumbar Scoliosis in a Patient With Proteus Syndrome: A Case Report and Literature Review

**DOI:** 10.1097/MD.0000000000000360

**Published:** 2015-02-06

**Authors:** Zheng Li, Jianxiong Shen, Jinqian Liang

**Affiliations:** From the Department of Orthopaedic Surgery (ZL, JS, JL), Peking Union Medical College Hospital, Chinese Academy of Medical Sciences and Peking Union Medical College, Beijing, China.

## Abstract

The Proteus syndrome (PS) is a complex and rare congenital hamartomatous condition with a wide range of malformations. Little is reported about spinal deformity associated with this syndrome.

This study presents a case of scoliosis occurring in the setting of PS and explores the possible mechanisms between the 2 diseases.

The patient is a 17-year-old Chinese female with scoliosis and hemihypertrophy of the right upper and lower extremity as well as exostosis of the right lower leg joint including the hip, knee, ankle, and toes. These manifestations were suggestive of PS. She underwent a posterior correction at thoracic 2-lumbar 4 (T5–L4) levels, using the Moss-SI spinal system. At 3-month follow-ups, the patient was clinically pain free and well balanced. Plain radiographs showed solid spine fusion with no loss of deformity correction.

The severity of scoliosis in PS is progressively aggravated and the correction of the extensive spinal deformities is generally difficult. Therefore, early diagnosis is required for adequate interdisciplinary treatment.

## INTRODUCTION

Proteus syndrome (PS) is a complex, rare, and variable disorder characterized by the patchy or segmental overgrowth of multiple tissues and organs including connective tissue nevi, epidermal nevi, vascular and lymphatic malformations, and craniofacial hyperostosis along with susceptibility to tumors.^1–3^ This syndrome was first described by Cohen and Hayden in 1979.^4^ Dietrich et al^5^ titled the disease, which has multiple, diverse, somatic manifestations, as the Greek god Proteus, who could change his form at will. The diagnostic criteria of the disease are yet to be properly established, and it may be confused with other similar syndromes such as Klippel–Trenaunay–Weber syndrome, neurofibromatosis, and Mafucci syndrome.^3,6^ About 200 cases of PS have been reported in the literature, and incidence of bone malformations is unknown.^7,8^ The exact cause, pathogenesis, and embryologic origin of PS remain a subject of discussion.^9,10^ There are limited reports regarding the diagnosis and management of PS with its possible resultant scoliosis. We here present a case of PS in a 17-year-old girl with unusual presentation of scoliosis.

## CONSENT

Written informed consent was obtained from the patient's parents on behalf of the child for publication of this case report and any accompanying images. A copy of the written consent is available for review by the Editor of this journal.

## CASE REPORT

**FIGURE 1:**
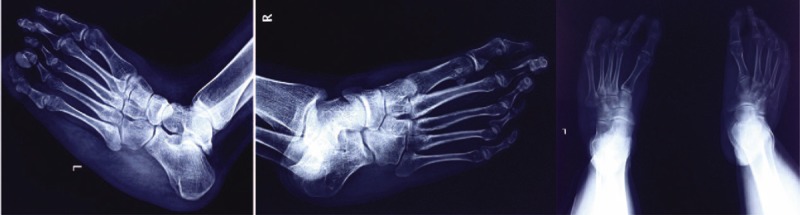
Standing anteroposterior and lateral radiographs of the preoperation.

A 17-year-old asylum seeker was admitted for an elective correction of her progressive scoliotic deformity. She was found to have scoliosis at age 3 by her parents. The brace therapy was initiated around 3 years of age, and the patient was compliant with the brace for 12 years, wearing it approximately 18 hours a day. There was also a history of progression of scoliosis despite brace therapy with thoracolumbosacral orthosis. Therefore, she was suggested the surgical correction for her spinal deformity. After 2 years, she was referred to the Peking Union Medical College Hospital, Beijing, China. Her plain radiographs of the spine showed that the Cobb angle of the thoracolumbar scoliosis was 100° and the Cobb angle of compensatory thoracic curvature was 70° (Figure 1), suggesting the need for surgical correction.

**FIGURE 2:**
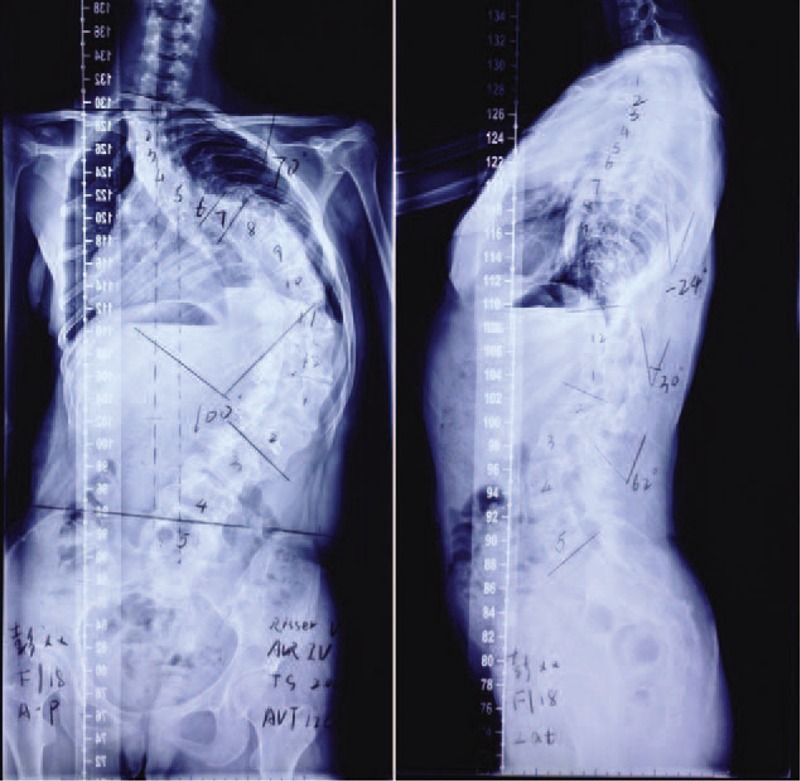
Radiograph of the foot showing enlargement of the first tarsometatarsal and metatarsophalangeal joints, with epiphyseal osteocartilaginous exostosis. Note the same finding in the fifth metatarsophalangeal joint.

Her past medical history was only remarkable in that she underwent resection of a soft tissue mass on the right side of her anterior abdomen at the age of 7. On physical examination, she has gradually demonstrated hemihypertrophy of the right upper and lower extremity as well as exostosis of the right lower leg joint including the hip, knee, ankle, and toes (Figure 2). She also showed increased thickness of the right plantar surface without cerebriform masses. For these asymmetrical deformities and joint dysfunctions, PS eventually was diagnosed. Magnetic resonance imaging revealed no evidence of any spinal cord or canal abnormalities. Computed tomography revealed no vertebral body deformities. The family history was unremarkable, and there was no parental consanguinity.

**FIGURE 3:**
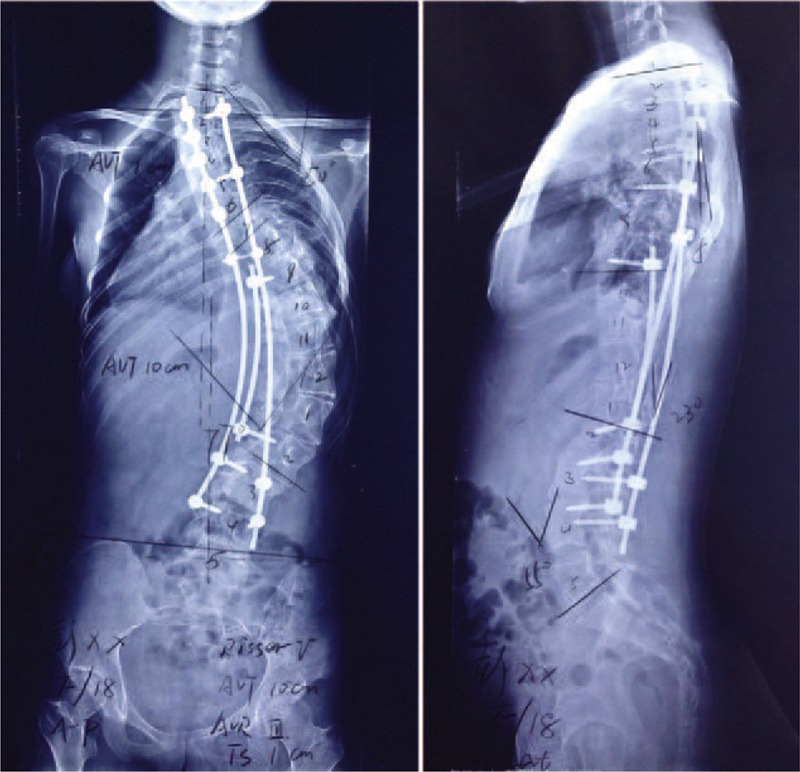
Standing anteroposterior and lateral radiographs of 4 d after operation.

In June 2014, a posterior correction and fusion at T2–L4 levels was performed, using the Moss-SI spinal system. The total operation time was 4 hours and 45 minutes. Total amount of blood loss was 800 mL. During the operation, the signal of this patient was normal using intraoperative spinal cord monitoring. Postoperatively, there was no sign of renal dysfunction. Postoperative plain x-ray film demonstrated the Cobb angles of the thoracolumbar scoliosis correction from 100° to 77° (correction rate 33%) (Figure 3). Her follow-up was asymptomatic, well balanced in the sagittal and coronal planes, with solid fusion at the third postoperative month. Both the patient and her parents were satisfied with the results of the surgery.

## DISCUSSION

PS is a group of disorders marked by partial gigantism of the hands, feet, or both; plantar hyperplasia; hemangiomas; lipomas; lymphangiomas; varicosities; verrucous epidermal nevi; macrocephaly; cranial exostosis; and asymmetry of the limbs because of long bone overgrowth.^11,12^ However, there are limited reports regarding the diagnosis and management of PS with scoliosis. In the present study, we reported a case of a 17-year-old female with scoliosis.

The diagnosis of PS is based on the existence of at least 4 of the major 7 criteria described by Samlaska et al^13^ in 1989. The diagnostic criteria for PS are summarized in Table 1.^14^ Despite these criteria, PS was often confused with a number of other syndromes with related disproportional skeletal enlargement including the following: hemihyperplasia syndromes, epidermal nevi (CLOVE) syndrome, type 2 segmental Cowden syndrome, neurofibromatosis, Klippel–Trenaunay–Weber syndrome, multiple enchondromatosis (Ollier disease and Maffucci syndrome), and Bannayan–Zonana syndrome.^11^ However, each of these syndromes can be distinguished from PS by meticulous observation of these characteristics.^3^ Scoliosis is a common manifestation of PS, which can range from one single long, gentle curve to multiple, severe curves.^6^ While the time of onset of scoliosis in PS is similar to that of adolescent idiopathic scoliosis, the severity and rate of progression in PS can be remarkable. There are no specific guidelines for scoliosis operations on patients with PS, but doctors must keep in mind that these PS including overgrowth of bones of the external auditory canal can cause conductive hearing loss, infection, and cholesteatomas.

**TABLE 1:**
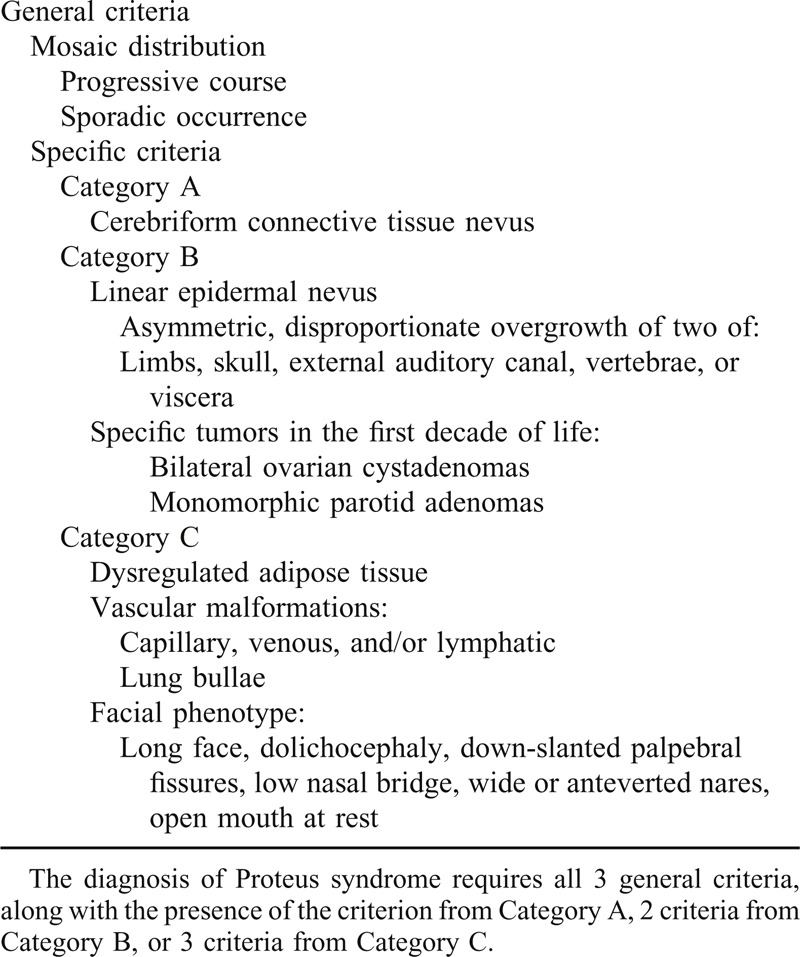
Diagnostic Criteria for Proteus Syndrome

The exact etiology, pathogenesis, and embryologic origin of PS syndrome are still not known.^1,15^ It is hypothesized to be caused by a new, mosaic, mutation acquired early in development; cells derived from the mutated cell line carry this mutation and result in affected tissues.^16,17^ For this patient, we propose that the localized overgrowth of the right lumbar facets and pedicles were the result of hemihypertrophy based on PS, not the influence of mechanical stress.

## CONCLUSION

In conclusion, PS is a relatively new rare syndrome described in recent years. The management of patients with PS and scoliosis is challenging. However, the cause of scoliosis in PS remains to be investigated. As the number of cases increases, the etiology, clinical manifestations, and natural history of this syndrome will become clearer. The severity of scoliosis in PS is progressively aggravated, and that correction of the extensive spinal deformities is generally difficult. Considering these findings, early diagnosis and treatment is recommended.
